# Validation of Orthopedic Postoperative Pain Assessment Methods for Dogs: A Prospective, Blinded, Randomized, Placebo-Controlled Study

**DOI:** 10.1371/journal.pone.0049480

**Published:** 2012-11-16

**Authors:** Pascale Rialland, Simon Authier, Martin Guillot, Jérôme R. E. del Castillo, Daphnée Veilleux-Lemieux, Diane Frank, Dominique Gauvin, Eric Troncy

**Affiliations:** 1 Groupe de Recherche en Pharmacologie Animale du Québec (GREPAQ), Department of Biomedical Sciences, Faculty of veterinary medicine, Université de Montréal, Saint-Hyacinthe, Quebec, Canada; 2 CiToxLAB North America, Laval, Quebec, Canada; 3 Department of Clinical Sciences; Faculty of veterinary medicine, Université de Montréal, Saint-Hyacinthe, Quebec, Canada; Imperial College London, United Kingdom

## Abstract

In the context of translational research, there is growing interest in studying surgical orthopedic pain management approaches that are common to humans and dogs. The validity of postoperative pain assessment methods is uncertain with regards to responsiveness and the potential interference of analgesia. The hypothesis was that video analysis (as a reference), electrodermal activity, and two subjective pain scales (VAS and 4A-VET) would detect different levels of pain intensity in dogs after a standardized trochleoplasty procedure. In this prospective, blinded, randomized study, postoperative pain was assessed in 25 healthy dogs during a 48-hour time frame (T). Pain was managed with placebo (Group 1, n = 10), preemptive and multimodal analgesia (Group 2, n = 5), or preemptive analgesia consisting in oral tramadol (Group 3, n = 10). Changes over time among groups were analyzed using generalized estimating equations. Multivariate regression tested the significance of relationships between pain scales and video analysis. Video analysis identified that one orthopedic behavior, namely ‘Walking with full weight bearing’ of the operated leg, decreased more in Group 1 at T24 (indicative of pain), whereas three behaviors indicative of sedation decreased in Group 2 at T24 (all p<0.004). Electrodermal activity was higher in Group 1 than in Groups 2 and 3 until T1 (p<0.0003). The VAS was not responsive. 4A-VET showed divergent results as its orthopedic component (4A-VETleg) detected lower pain in Group 2 until T12 (p<0.0009), but its interactive component (4A-VETbeh) was increased in Group 2 from T12 to T48 (p<0.001). Concurrent validity established that 4A-VETleg scores the painful orthopedic condition accurately and that pain assessment through 4A-VETbeh and VAS was severely biased by the sedative side-effect of the analgesics. Finally, the video analysis offered a concise template for assessment in dogs with acute orthopedic pain. However, subjective pain quantification methods and electrodermal activity need further investigation.

## Introduction

Postoperative pain remains the main cause of morbidity related to surgery. Spontaneous nociceptive pain has been associated with both skin incisions [1] and deep surgery [2]. Using a rodent knee surgery model, Buvanendran *et al.* [3] also characterized some functional (behavioral) outcomes. In addition, nociceptive stimulation and neuronal changes might differ between those observed in models of acute postsurgical pain [2] and chemical models of acute inflammation like sodium urate-induced synovitis in dogs [4], [5], [6]. In this context, a standardized and technically well-recognized canine orthopedic surgery might be a stronger surrogate of surgical pain than a chemically inflammatory pain model in dogs. Altogether, rodent models and chemical models would present a limited approach to the complex process of pain associated with orthopedic surgery. Veterinary surgeons, on the other hand, manage many natural painful disease processes that are common to both dogs and human beings [7], and they perform preclinical and clinical orthopedic procedures in dogs, some of which are directly derived from procedures used in human beings [8]. Consequently, we argue that common orthopedic dog surgeries, as trochleoplasty, are valid surrogates for the investigation of human surgical pain [9], [10]. However, methods of pain assessment in dogs are not extensively documented or standardized [11], [12], [13]. Inadequate pain assessment for dogs decreases the validity of canine pain models and hampers the comparison of pain studies.

Both owners [14] and veterinarians [15], [16] associate orthopedic surgery with a high degree of pain. Behavioral (guarding, interaction with owner, reaction to palpation, etc.) and physiological (cardiovascular indices and stress response) indicators are commonly used to assess pain in non-verbal patients [17]. Composite pain scales and multidimensional questionnaires have been developed for use in a wide range of canine postoperative pain conditions [11], [12], [13]. According to the Cohen’s classification [18], both postoperative pain scales Glasgow Composite Pain Scale and University of Melbourne Pain Scale were developed in compliance with the psychometrics rules. However, these instruments did not differentiate the analgesic effect of the standard non-steroidal anti-inflammatory drugs (NSAID) from this of the Coxib [19] or fentanyl [20] in pain studies with canine orthopedic surgery. In contrast, kinetic gait analysis using a force plate or a pressure-sensing weight mattress decreased following sodium urate-induced acute synovitis in dogs [21], [22] and improved following NSAID drug administration [21], [23], [24]. Kinetic gait analysis did no correlate well with the subjective lameness scoring in dogs [25], [26], [27], [28], which supported the kinetic analysis to be a more sensitive indicator of joint pain than subjective lameness scoring. Even if kinetic gait analysis is a great asset in lameness study, it might not capture the broader aspects of pain [29] and is not available in every clinical center. Recently, the behavioral assessment of rodent pain has evolved with the use of semi-automated behavioral video analysis [30] and standardized behavioral facial expression coding systems [31]. Previously, video analysis of spontaneous behaviors in dogs after ovariohysterectomy allowed unique discrimination between pain-related behaviors and drug side effects, such as sedation [32], [33], but this pain monitoring method has not yet been used following canine orthopedic surgery.

The purpose of the present study was to evaluate methods of pain assessment following canine orthopedic surgery. The hypothesis was that the effects of three different levels of analgesia would be reflected and therefore recognizable by the behavioral and physiological changes they elicit on a standardized canine postoperative pain model. Several methods (video analysis of spontaneous behaviors, electrodermal activity [EDA], visual analogue scale [VAS], and composite pain scale [4A-VET pain scale]) were used for the assessment of postoperative pain to evaluate their reliability and responsiveness. The concurrent validity of the behavioral pain assessment tools was tested using video analysis as the reference method.

## Results

### Rescue Analgesia

Rescue analgesia was provided for 25% of all dogs. Rescue analgesia requirements were distributed as follows: three dogs from Group 1 at Time (T)0.41, T6.01 and T6.31, one dog from Group 2 at T24.01, and two dogs from Group 3 at T1.19 and T6.19 hours postoperatively, with T0 being the extubation time.

### Video Analysis of Spontaneous Behaviors

Of all the spontaneous behaviors identified in our ethogram (Appendix S1), fourteen that occurred frequently were statistically analyzed and their reliability tested (Table 1). Eleven behaviors presented a moderate to high inter-observer reliability, as their intraclass correlation coefficient ranged from 0.50 to 0.99.

**Table 1:** Interobserver reliability of the video-analysis.

Spontaneous behaviour Observer	Number #1	Number #2	Mean (SD) #1	Mean (SD) #2	ICC
Standing with no weight bearing	6	4	4.3 (3.6)	6.7 (3.6)	**0.74**
Walking with full weight bearing	4	3	136.2 (141.9)	54.6 (30.1)	**0.99**
Standing with full weight bearing	4	3	83.0 (105.8)	67.0 (93.5)	**0.97**
Sitting normal with equal weight on limbs	4	3	11.7 (15.0)	7.3 (3.5)	**0.84**
Immobile with head down	4	3	49.5 (24.7)	17.0 (10.0)	**0.52**
Silent	5	5	63.2 (49.0)	46.8 (48.7)	0.01
Howling	5	5	63.4 (51.8)	24.0 (41.5)	**0.50**
Sniffing	8	8	182.1 (138.8)	87.0 (47.7)	**0.98**
Immobile with head up	8	8	167.1 (123.2)	31.3 (17.3)	**0.91**
Immobile while looking around	8	8	32.2 (23.7)	34.0 (21.07)	0.39
Licking lips	6	6	50.8 (51.9)	19.8 (20.1)	**0.92**
Ears twitching	6	5	6.1 (3.6)	4.6 (2.6)	0.01
Ears normal	8	8	69.1 (47.8)	25.5 (15.5)	**0.87**
Dog in front of the kennel	8	8	34.7 (28.2)	36.3 (30.5)	**0.99**

Data presents the number of videotapes (Number) for which the behaviour was recorded by both independent observers (#1 and #2) blinded to treatment groups for 10% of all videos (n = 8). Mean (SD) of the occurrence rate is presented for each observer (#1 and #2). Intraclass correlation coefficients (ICC) were calculated from a set of 10% randomized videotapes.

For ‘Walking with full weight bearing’, ‘Howling’, ‘Sniffing’, and ‘Licking lips’, generalized estimating equation (GEE) analysis indicated significant main effects for time and group, as well as a significant interaction between group and time (Table 2). For the occurrence rates of ‘Standing with full weight bearing’, and ‘Dog in front of the kennel,’ there was no group effect, but there were significant effects of time and a significant interaction between group and time (Table 2). There were no significant interactions between group and time for the remaining behaviors, thus explaining why they were discarded from further analyses.

**Table 2:** Wald statistics for Type 3 GEE^1^ analyses of video-analysis.

Spontaneous behaviour	Time ChiSq (p)	Group ChiSq (p)	Group × Time ChiSq (p)
Standing with no weight bearing	198.91 (<.0001)	35.8 (<.0001)	5.77 (0.21)
**Walking with full weight bearing**	**920.21 (<.0001)**	**16.15 (0.0003)**	**33.57 (<.0001)**
***Standing with full weight bearing***	***1692.66 (<.0001)***	***4.27 (0.12)***	***11.47 (0.022)***
Sitting normal with equal weight on limbs	996.06 (<.0001)	2.23 (0.33)	5.36 (0.25)
Immobile with head down	3214.42 (<.0001)	4.72 (0.09)	3.61 (0.46)
**Howling**	**1800.35 (<.0001)**	**7.1 (0.03)**	**170.58 (<.0001)**
**Sniffing**	**4141.17 (<.0001)**	**19.74 (<.0001)**	**9.49 (0.05)**
Immobile with head up	4564.48 (<.0001)	10.34 (0.006)	6.4 (0.17)
**Licking lips**	**834.88** (<.0001)	**17.76 (0.0001)**	**29.71 (<.0001)**
Ears normal	1485.69 (<.0001)	3.05 (0.22)	2.49 (0.65)
***Dog in front of the kennel***	***2745.23 (<.0001)***	***0.75 (0.69)***	***36.02 (<.0001)***

1 Generalized estimating equation. For each behaviour, the results of GEE analysis are presented as the Chi-square result (ChiSq) and the p-value (p) of the main effect for Time, Group and the Group × Time interaction. Significant main effect for Group × Time interaction is presented in bold. Italics for the behaviours indicated in bold is indicative of no significant Group effect.

The planned comparison analysis over time indicated that the occurrence rates of the remaining behaviors (as listed in Table 3) were not different across groups at baseline: T-96 (all *p*>0.08). From T-96 to T24, the occurrence rate decreased for ‘Walking with full weight bearing’, ‘Standing with full weight bearing’, ‘Howling’, and ‘Sniffing’ in all treatment groups (all *p*<0.0001, Table 3). Furthermore, for ‘Licking lips’ and ‘Dog in front of the kennel’, the occurrence rates declined in Group 2 (*p*<0.0001, and *p*<0.0001, respectively) and Group 3 (*p* = 0.001, and *p* = 0.0002, respectively) from T-96 to T24 (Table 3) but did not change in Group 1 (*p* = 0.69, and *p* = 0.09, respectively).

**Table 3:** Descriptive statistics of spontaneous behaviour during video-analysis.

Spontaneous behaviour	Group	T-96 Med (min-max) Freq (%)		T24 Med (min-max) Freq (%)		T48 Med (min-max) Freq (%)	
**Walking with full weight bearing**							
	1	186 (40–308)	***16.9^x^***	0 (0–2)	***0.05^a^***	64 (0–228)	*10.78*
	2	139 (9–321)	***27.64^x^***	0 (0–3)	***0.12^a^***	1 (0–109)	*6.94*
	3	250 (81–332)	***24.25^x^***	3 (0–117)	***2.4^b^***	6 (0–145)	*4.81*
**Standing with full weight bearing**							
	1	52(7–128)	***6.02^x^***	4 (0–25)	*0.57*	22 (0–103)	*3.69*
	2	45 (5–179)	***11.84^x^***	4 (0–4)	*0.48*	3 (0–21)	*1.63*
	3	57 (25–304)	***11.22*** *^x^*	4 (0–10)	*0.42*	6 (0–24)	*0.74*
**Howling**							
	1	1 (0–192)	***2.25^x^***	0 (0–33)	***0.58^a^***	3 (0–73)	*1.38*
	2	13 (0–111)	***8.48^x^***	0 (0–1)	***0.04^b^***	0 (0–4)	*0.25*
	3	21 (0–115)	***4.02^x^***	1 (0–13)	*0.40* ***^ab^***	2 (0–43)	*0.72*
**Sniffing**							
	1	208 (78–373)	***23.86^x^***	89 (17–213)	*9.49*	178 (8–395)	*21.47*
	2	147 (98–295)	***37.92^x^***	10 (0–93)	*4.76*	77 (9–130)	*18.25*
	3	301 (112–483)	***31.06^x^***	124 (1–269)	*13.38*	98 (1–374)	*12.27*
**Licking lips**							
	1	7 (0–23)	*1.12*	7 (0–52)	***1.42^a^***	17 (0–72)	***2.88^a^***
	2	10 (2–116)	***5.80^x^***	1 (0–1)	***0.12^b^***	3 (0–5)	***0.63^b^***
	3	10 (7–11)	***2.80^x^***	7 (0–30)	***0.96^a^***	18 (0–68)	***2.54^a^***
**Dog in front of the kennel**							
	1	28 (6–130)	*3.98*	9 (2–72)	***2.12^a^***	23 (4–57)	*3.47*
	2	27 (2–181)	***10.68^x^***	2 (1–12)	***0.84^b^***	18 (1–85)	*7.63*
	3	78 (12–219)	***9.32^x^***	7 (1–55)	*1.79* ***^ab^***	21 (1–75)	*2.20*

Data are presented as the median (Med), minimum (min), maximum (max) and relative frequency (Freq) in percentage (%) of spontaneous behaviour by group and time (T) −96, 24 and 48 hours. Superscript case (x): significant difference when −96 h is compared to 24 h; At each time point, different letters (higher case (a) or (b)) indicate significantly different values among treatment groups. Significant differences are presented in bold. Bonferroni-corrected alpha level was of 0.0041.

At T24, the estimated occurrence of ‘Walking with full weight bearing’ was 43.3 (95% confidence interval [95%CI]: 11.1, 170.7; *p*<0.0001), and 36.2 (95%CI: 4.5, 285.7; *p* = 0.0007) times higher in Group 3 than in Groups 1 and 2, respectively (Table 3). Also at T24, the estimated occurrences of ‘Howling’, ‘Licking lips’, and ‘Dog in front of the kennel’ were 29.0 (95%CI: 3.5, 235.4; *p* = 0.001), 23.6 (95%CI: 8.6, 65.0; *p*<0.0001), and 7.7 (95%CI: 3.1, 18.9; *p* = 0.003) times higher, respectively, in Group 1 than in Group 2. Moreover, the observed difference between Groups 1 and 2 for ‘Licking lips’ persisted at T48 with an estimated risk ratio of 10.4 (95%CI: 3.9, 27.8; *p*<0.0001) (Table 3). The occurrence of ‘Licking lips’ was also higher in Group 3 than in Group 2 both at T24 and T48, with estimated risk ratios of 13.9 (95%CI: 5.1, 38.3; *p*<0.0001) and 10.9 (95%CI: 4.1, 28.8; *p*<0.0001), respectively (Table 3).

### Electrodermal Activity

**Figure 1:**
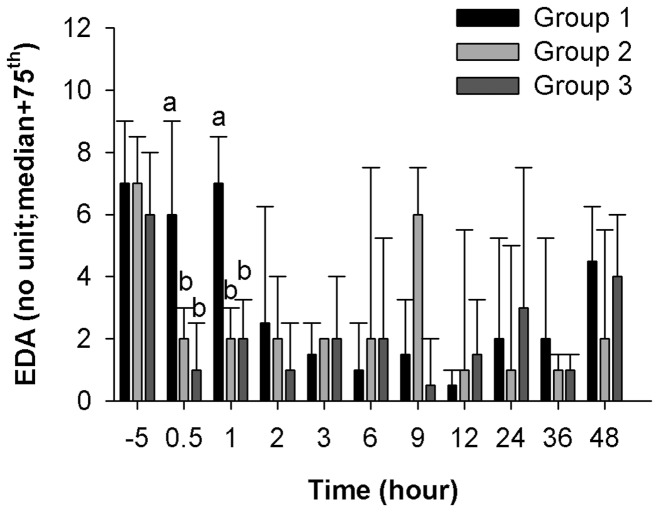
Electrodermal activity. EDA (no unit) by group over time. Data are presented as the median and 75^th^ percentile for groups of n = 5 to 10 dogs over time. At each time point, different letters (higher case (a) or (b)) indicate significantly different values among treatment groups. Bonferroni-corrected alpha level was of 0.0015.

The EDA measurements at T-96 were not correlated with those at T-5 (Spearman’s rank correlation (rho_s_) = 0.001, *p* = 0.87), and Cohen's kappa coefficient (κ) could not be computed. The EDA measurement analysis indicated an overall time effect (*p*<0.0001), and a significant interaction between group and time (*p*<0.0001), but there was no significant group effect (*p* = 0.40). The planned comparisons showed that the EDA measurements of Group 1 were higher than those of Groups 2 and 3 at T0.5 (*p*<0.0001, and *p*<0.0001, respectively) and T1 (*p*<0.0001, and *p* = 0.0003, respectively) (Figure 1).

### Pain Scales

The VAS reliability was not estimated, because all of the scores were 0 at T-96 and T-5. For the composite pain scale, namely 4A-VET pain scale, scores at T-96 and T-5 were correlated (rho_s_ = 0.52; *p* = 0.008) and demonstrated fair agreement (κ = 0.33, 95%CI: 0.08, 0.57) [34]. Cronbach's alpha coefficient was 0.7, indicating that the items of the 4A-VET pain scale were homogeneous.

**Figure 2:**
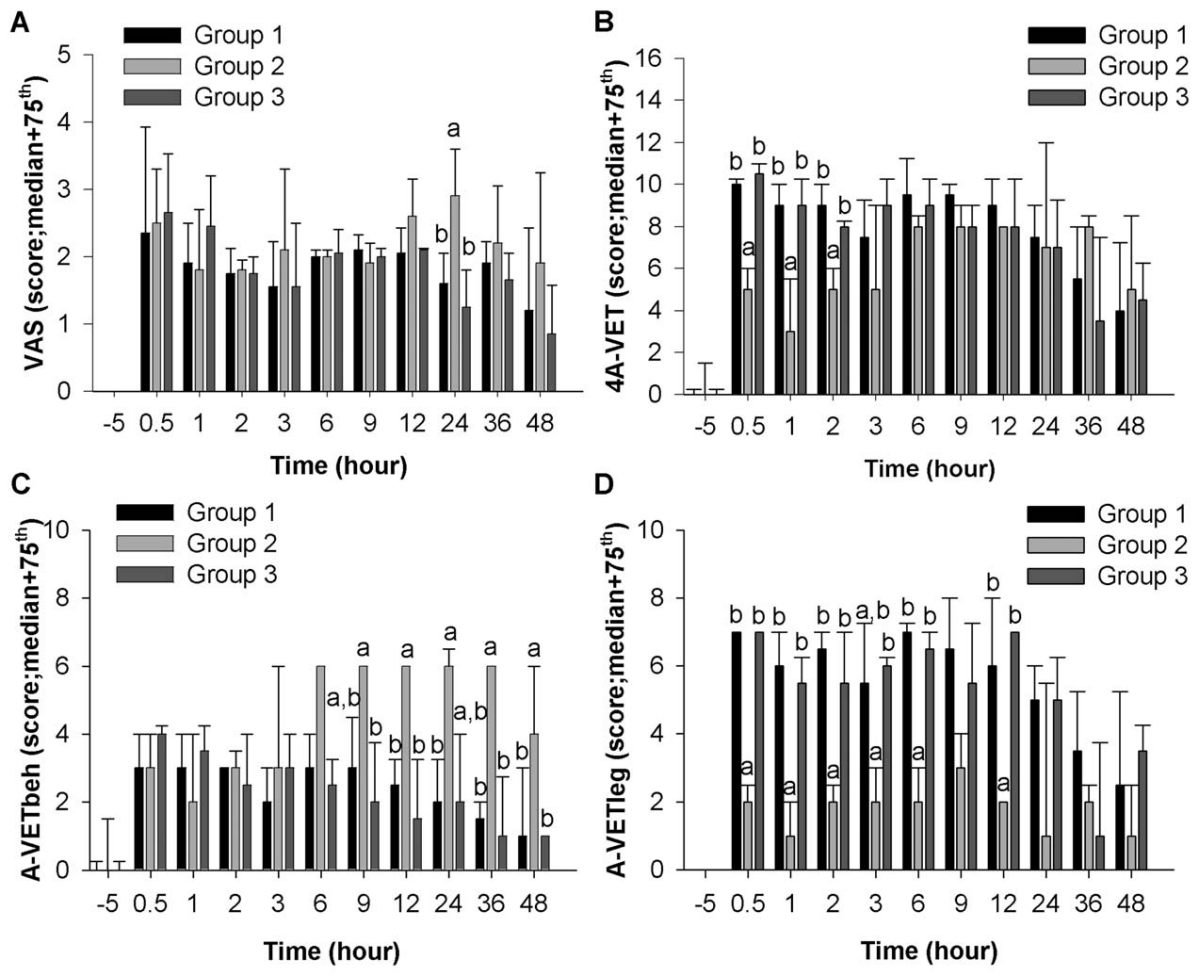
Pain scales. A) Visual analogue scale, **B)** the 4A-VET pain scale, **C)** 4A-VETbeh subscale, and **D)** 4A-VETleg subscale by group over time. Data are presented as the median and 75^th^ percentile for groups of n = 5 to 10 dogs at each time points. At each time point, different letters (higher case (a) or (b)) indicate significantly different values among treatment groups. Bonferroni-corrected alpha level was of 0.0015.

An analysis of the VAS scores indicated overall effects of time (*p*<0.0001) and group (*p* = 0.003), as well as a significant interaction between group and time (*p*<0.0001). At T24, Group 2 presented higher pain scores than Groups 1 and 3 (*p*<0.0001, and *p*<0.0001, respectively) (Figure 2A).

An analysis of the 4A-VET scores indicated overall effects of time (*p*<0.0001) and group (*p*<0.0001), as well as a significant interaction between group and time (*p*<0.0001). Group 2 presented lower scores than Groups 1 and 3, at T0.5 (both *p*<0.0001), T1 (both *p*<0.0001) and T2 (*p* = 0.0009, and *p* = 0.0012, respectively) (Figure 2B).

An analysis of the behavioral component of 4A-VET (4A-VETbeh) scores showed overall effects of time (*p*<0.0001) and group (*p*<0.0003), as well as a significant interaction between group and time (*p*<0.0001). Group 2 had higher 4A-VETbeh scores than Groups 1 and 3, at T12 (*p* = 0.0001, and *p* = 0.0003, respectively), T36 (*p* = 0.0002, and *p* = 0.0003, respectively), and T48 (*p* = 0.0011, and *p*<0.0001, respectively) (Figure 2C). In addition, the 4A-VETbeh scores of Group 2 were higher than those of Group 3 at T9 (*p* = 0.0015) and those of Group 1 at T24 (*p* = 0.0002) (Figure 2C).

An analysis of the orthopedic component of 4A-VET (4A-VETleg) scores indicated main effects of time (*p*<0.0001) and group (*p*<0.0001), as well as a significant interaction between group and time (*p*<0.0001). Group 2 presented significantly lower 4A-VETleg scores than Groups 1 and 3, at T0.5 (both *p*<0.0001), T1 (both *p*<0.0001), T2 (*p* = 0.0002, and *p* = 0.0009, respectively), T6 (*p*<0.0001, and *p* = 0.0002, respectively), and T12 (both *p*<0.0001) (Figure 2D). Additionally, the 4A-VETleg scores of Group 2 were lower than those of Group 3 at T3 (*p* = 0.0008) (Figure 2D).

### Concurrent Validity

A multivariate GEE regression analysis indicated that all of the pain scores were negatively associated with the administration of rescue analgesia (β estimate±SE: −0.92±0.30; *p* = 0.002 for VAS, −2.19±0.58; *p* = 0.0002 for 4A-VET, −2.20±1.04; *p* = 0.04 for 4A-VETbeh, and −0.92±0.30; *p* = 0.002 for 4A-VETleg). Except for 4A-VETbeh, all pain scores were positively associated with time (0.79±0.14; *p*<0.0001 for VAS, 0.89±0.29; *p* = 0.002 for 4A-VET, and 0.79±0.14; *p*<0.0001 for 4A-VETleg). In addition, VAS scores were negatively associated with the occurrence of two behaviors: ‘Walking with full weight bearing on the operated limb’ (−0.13±0.04; *p* = 0.0003) and ‘Dog in front of the kennel’ (−0.14±0.06; *p* = 0.03), while 4A-VET scores were negatively associated with ‘Walking with full weight bearing’ (−0.22±0.03; *p*<0.0001). 4A-VETbeh scores were positively associated with ‘Immobile with head down’ (0.42±1.04; *p* = 0.02) and negatively associated with ‘Sniffing’ (−0.43±0.10; *p*<0.001). Finally, 4A-VETleg scores were positively associated with ‘Standing with no weight bearing on the operated limb’ (0.73±0.25; *p* = 0.004) and negatively associated with both ‘Walking with full weight bearing’ (−0.20±0.06; *p* = 0.002) and ‘Sitting normally with equal weight on limbs’ (−0.57±0.26; *p* = 0.04).

## Discussion

In this model, the hypothesis was that Group 1 would present the most pain, Group 2 the least, and Group 3 intermediate pain. The results partially support this hypothesis although no behavioral or physiological assessment demonstrated the expected gradient of pain response (Group 1>Group 3>Group 2). Group 1 did present more pain than the other two groups, as demonstrated clearly by one video analysis criterion, namely ‘Walking with full weight bearing’ (Group 1 ≠ Group 3, at T24), as well as by 4A-VETleg (Group 2 ≠ Groups 1 and 3, at T0.5, 1, 2, 3, 6, and 12) and EDA (Group 1 ≠ Groups 2 and 3, at T0.5, and 1). Heavy sedation appears to explain the lack of specificity in pain detection by the other methods (namely VAS and 4A-VETbeh). Most interestingly, the multimodal analgesia group (#2) was higher than would be expected for VAS at T24 and for 4A-VETbeh at T9, 12, 24, 36, and 48. This was definitively a concurring and surprising discovery. Inclusion of anesthesia/drug controls would have assisted in determining the effects of sedation. In fact, video analysis confirmed that analgesic drug-induced sedation decreased some behaviors because dogs in Group 2 spent less time acting interested to their environment (‘Dog in front of the kennel’), or trying to attract attention (‘Howling’), and making facial expressions (‘Licking lips’). With regards to our specific objectives, we observed good reliability for eleven behaviors in the video analysis (see Table 1) and for the 4A-VET pain scale. We could not evaluate VAS reliability, and EDA reliability was poor. Establishing measurement reliability was an obligatory step before we could assess responsiveness and concurrent validity.

In animal video analysis, there are numerous methods for recording behavioral changes. In this study, we performed a microanalysis approach of events based on a quantitative description of an animal’s normal behavior. The method generated a wide range of behaviors and occurrences. Only behaviors that demonstrated significant occurrence rates and high inter-observer reliability were selected as final endpoints. This selection method could be considered quite limiting, particularly as the duration of video was a one hour-period, and the inter-observer reliability was tested on 10% of randomized videotapes. We deemed that these strict behavioral criteria would be strongly representative of postsurgical orthopedic pain.

A decreased occurrence rate of ‘Walking with full weight bearing’ was demonstrated in all treatment groups following trochleoplasty compared to normal behavior. At T24, this decrease was higher in Group 1 than in Group 3, suggesting that tramadol in the latter group provided some analgesia-related use of the operated limb. Intuitively, it makes sense to measure the occurrence rate of ‘Walking with full weight bearing on the operated limb’ as a measure of orthopedic pain (or, at least as a measure of an absence of lameness) but no previous study has investigated this measurement as an indicator of pain.

So far, it was postulated that the degree of pain would correlate to the degree of weight bearing using force plate systems. Unexpectedly, the occurrence rate of the spontaneous behavior ‘Standing/walking/trotting with no or partial weight bearing’ (as indicator of lameness) did not discriminate different levels of pain and indicated the lack of specificity of this measurement using video-analysis. It is also possible that the observer-reported behavior was less accurate in quantifying lameness than evaluating absence of lameness in dogs. This hypothesis would be in accordance with previous publications reporting a lack of correlation between subjective lameness scores and weight bearing measurements recorded through kinetic gait analysis in canine studies [25], [26], [27], [35]. Nevertheless, postoperative pain was correlated to a decrease in the occurrence rate of a normal behavior ‘Walking with full weight bearing of the operated leg’, suggesting that this latter behavior was a specific pain-free behavior. Our result supported that first, painful dogs were less active (walk, trot); second, the dogs were either lame or not lame when they were active; and third, the naturally occurring behavior of severity of lameness was not correlated to pain severity using video-analysis. Altogether, recording of a spontaneous behavior should not be interpreted in the same way as kinetic gait analysis. Kinetic gait analysis was currently performed when dogs were compelled to walk or trot, suggesting a sustained nociceptive firing during limb use. Indeed, video-analysis would summarize the way the dogs behaved and responded to postoperative pain, suggesting a cognitive adaptation to pain. Therefore the sensitivity of the behavioral quantification of ‘Walking with full weight bearing of the operated leg’ supports its use for further study as a new surrogate for assessing pain in clinical conditions.

‘Howling’ frequency differentiated Group 2 from Group 1 at T24, and the difference between Groups 2 and 3 approached significance (*p* = 0.006– Table 3). It is generally acknowledged that increased vocalization is associated with postoperative pain expression in the dog as reflected by the inclusion of this behavior in many canine postoperative pain scales [13], [36], [37]. However, a decrease in ‘Howling’ in all three groups could indicate that postoperative pain decreased the occurrence rate of ‘Howling’, particularly for Group 1. That the decrease was more pronounced in Group 2 suggests that the use of a multimodal analgesic protocol may have contributed more to the decrease than did pain. Indeed, the occurrence rate did not return to its baseline value in any group. This finding supports vocalization’s lack of sensitivity to postoperative pain intensity, as was observed in a previous study [38].

The occurrence rates of both ‘Dog in front of the kennel’ and ‘Licking lips’ behaviors decreased in Group 2 when compared to Group 1 and decreased in both analgesic groups over time, while remaining stable over the same period in Group 1. Moreover, Group 2 spent less time ‘Licking lips’ during the overall postoperative period. Altogether, the decreased occurrence rate observed in both pharmacologically treated groups, and mostly in Group 2, may simply not be related to a pain-controlling effect but rather may be related to a sedative effect of the different opioids (fentanyl patch, epidural morphine, and oral tramadol). Similar results were previously observed following administration of butorphanol in a canine pain study [32]. These results highlight the major interference of the neuropharmacological effects of commonly used analgesic (opioid) drugs in the apparent expression of postoperative pain. The frequency of these two behaviors did not change over time in Group 1, suggesting that they were not affected by postoperative pain. Thus, the observations regarding restlessness/interest in the environment indicated by ‘Dog in front of the kennel’ and ‘Licking lips’ behaviors should be analyzed with caution and may not demonstrate assay sensitivity for comparing analgesic protocols.

Altogether, video analysis was a powerful method that provided evidence of pain related behaviors and identified behaviors related to drug side-effects. The low number of selected and validated spontaneous behaviors is related to inter-subject variability in pain expression and to the difficulty associated with standardizing a behavioral observation for assessing pain. These results support the use of video analysis as a valid pain assessment tool because of its ability to test concurrent validity with subjective behavioral pain assessments. The concurrent validity analysis completed in this study confirmed sedation’s major influence not only on video analysis but also on VAS and 4A-VETbeh scores.

In this study, EDA and 4A-VET were responsive to multimodal analgesia in the immediate postoperative period by reporting decreased skin conductance, a known method for indirectly quantifying sympathetic activity and decreased (4A-VET) pain scores, respectively, in Group 2. There were slight discordant responses between EDA measurements and 4A-VET scoring. With EDA, Group 1 demonstrated higher pain intensity compared to Groups 2 and 3, whereas with 4A-VET, the intensity of pain was lower in Group 2 than in both Groups 1 and 3 at similar time points.

The most plausible explanation for the decreased EDA intensity in Groups 2 and 3 was the analgesia/anxiolysis induced by either treatment. This analgesic detection is supported by a study where the EDA intensity correlated significantly with kinetic gait analysis, telemetered motor activity and subjective scoring to demonstrate analgesic effect of a bisphosphonate in an experimental dog osteoarthritis model [39]. Hypothetically, the EDA decrease could also be related to other pharmacological interactions with sympathetic activity [40], [41], [42]. Moreover, the sensitivity of EDA was not important, as highlighted by the absence of a difference between Groups 2 and 3 and the short duration of its effectiveness to differentiate Group 1 from both Groups 2 and 3. This low psychometric quality added to the previously reported lack of specificity in a rodent model of surgical pain [43]. Further investigation is needed before considering increasing the clinical use of EDA.

Although other canine pain studies have validated the pain VAS [44], [45], different treatment effects on mean VAS scores following trochleoplasty were not demonstrated in this study. The VAS might have provided systematic error, particularly when measuring pain at baseline (floor effect) and during the postoperative period (ceiling effect). The VAS observer could not be blinded to the presence or the absence of surgery because sham dogs were not included, and therefore this was an evident first source of bias (explaining the floor effect). Furthermore, increased VAS scores in Group 2 at T24 suggested that sedative side-effects of analgesics might interfere with VAS scoring. Confounding effect of analgesic side effect on VAS score (increasing it) was previously observed [46], [47]. This could explain the lack of sensitivity in postoperative pain quantification using the VAS and the absence of a treatment effect using this method. Altogether, these findings urge for caution in the use and interpretation of observer-reported VAS pain scoring as a standardized pain assessment method with experimental animals as it could be biased and not specific for pain.

The 4A-VET pain scale showed acceptable reliability and, as reported earlier, was partially responsive to treatments. Nevertheless, like EDA, 4A-VET demonstrated weak performance because of its apparent low sensitivity (no difference between Groups 1 and 3) and short duration (initial 2 hours post-surgery) of effective responsiveness in favor of Group 2. Interestingly, the weak performance of the 4A-VET pain scale could be explained by the response divergence between its two main components, namely 4A-VETbeh and 4A-EVTleg. The 4A-VETleg scores indicated significantly lower pain in Group 2 compared to Groups 1 and 3 at T0.5, 1, 2, 3, 6, and 12. Conversely, 4A-VETbeh scores indicated increased pain for Group 2 compared to Groups 1 and 3 at T9, 12, 24, 36, and 48. Considered together, these results suggest that non-analgesic effects of the multimodal analgesia protocol used in Group 2 may have been a potential confounder in pain assessment, as has been observed in previous canine postoperative pain studies [38], [44]. The differences between the mean scores of 4A-VETbeh and 4A-VETleg might also illustrate differences in scale construction. This is a strong argument for choosing 4A-VETleg as a standard measure of orthopedic postoperative pain. Nevertheless, 4A-VETleg had some limitations because it was not as responsive at T24 as was the video analysis, suggesting that 4A-VETleg was valid during at least the first twelve hours following surgery. At this point, the validity of behavioral pain assessment based on pain scoring systems is uncertain because many questions remain in relation to measurement errors and the difficulty of weighing the consequence of sedation against those of unrelieved pain, as has already been observed [48].

Using video analysis, ‘Walking with full weight bearing’ of the operated limb was the only validated behavior to support the analgesic efficacy of tramadol as well as to indicate the presence of pain in Group 1. Analgesic side-effects strongly associated with behavioral changes. Regression methods were used to test the concurrent validity of the pain scales scores with video analysis as the standard of the behavioral pain assessment. Of all displayed behaviors, ‘Walking with full weight bearing on the operated limb’ was the behavior that was most correlated with the VAS, 4A-VET and 4A-VETleg pain scores. It is possible that the relationship between the pain scales and ‘Walking with full weight bearing’ occurred for several reasons: 1) this behavior was more frequent; 2) this higher frequency could be attributed to a more conservative and well-understood definition, allowing it to be observed with more accuracy; and 3) recording during daytime might have improved the robustness of the occurrence rate of ‘Walking with full weight bearing’ in relation to the dog’s level of daylight activity.

Additionally, it appears this behavior (‘Walking with full weight bearing’) is unconsciously linked to pain-free behavior for VAS and 4A-VETleg. Moreover, for the latter, pain intensity was clearly linked to lameness (reflected by ‘Standing with no weight bearing’), thus reinforcing the conceptual validity of 4A-VETleg scores. Interestingly, the regression analysis in this study confirmed the previously suspected limitations in the pain scoring systems. First, sedative side-effect of the drug(s) was a confounding factor for assessing pain with VAS because VAS was linked to the spontaneous behavior ‘Dog in front of the kennel’ that changed in response to the side-effect of the analgesic. Second, the regression models revealed that 4A-VETbeh scores were related to two spontaneous behaviors, ‘Immobile with head down’ and ‘Sniffing’, which were not validated by the video analysis and were assumed to be included in the communicative category. The video analysis confirmed that the present 4A-VETbeh was not an accurate method for pain evaluation in this study. The results also showed strong evidence that the large number of items in the composite 4A-VET pain scale introduces noise into this pain scoring system.

Pain expression may hypothetically differ when an animal is observed directly as opposed to being filmed without a person in the environment. This could, evidently, lead to differences in pain observation using various methods. The advantage of video analysis for pain expression is that it can be used as a reference method to introduce further development of pain scales [49], as has been previously performed in dogs [48]. It is important to consider that many factors can influence the measurement of pain. It has been proposed that not only the pain stimulus itself, but observer characteristics, environmental and social interaction effects, and intra-subject factors can all influence the measurement of pain. This can occur *via* effects on the pain experience, as well as on its expression [49]. In this study, the standardization of procedures allowed us to control all of these aspects, except the intra-subject experience.

An important limitation of the study was the apparent moderate intensity of postoperative pain generated by the trochleoplasty procedure, as reflected by the low levels of pain scale scoring, as well as the low use of rescue analgesia in Groups 1 and 3. This could be related to an inadequate sensitivity of pain scales. A higher intensity of pain would surely have contributed to better discrimination in pain assessment method responsiveness.

In conclusion, the video analysis provided strong evidence of responsiveness and validity of the 4A-VETleg pain scale for assessing acute orthopedic pain. The alteration of normal gait behavior, as observed by changes in ‘Walking with full weight bearing,’ is likely to be the best behavioral orthopedic pain assessment method. The current results will hopefully contribute to the generation of a refined and validated method of orthopedic pain assessment. This study also clearly establishes the major interference of analgesic side effects on dog behaviors. This is a major finding with regards to the use of opioid drugs as a staple in the surgical analgesic arsenal in veterinary and human medicine. Such interference could potentially contribute to the overdosing of opioids.

## Methods

### Ethics Statement, Animals and Experimental Design

The Institutional Animal Care and Use Committee approved the study protocol (# Rech-1220), and the Canadian Council on Animal Care guidelines were followed regarding care and handling of the dogs. This study also adhered to the guidelines of the Committee for Research, Ethical Issues of the IASP [17] and the ARRIVE checklist [50]. Twenty-five healthy male beagle dogs (15.2 (3.3) [mean (SD)] months old and weighing 9.9 (1.4) kg) belonging to the colony of a contract research organization (CiToxLAB North-America, Inc.) accredited by the Association for Assessment and Accreditation of Laboratory Animal Care International were included. Dogs were acclimated for 1 week and housed in individual kennels under standard laboratory conditions in a 12 h light/dark cycle with food and water provided *ad libitum*. Dogs were maintained in standard environmental conditions (humidity, temperature, and ventilation).

Baseline evaluations were carried out before surgery at –96 h (video analysis occurrence rate of spontaneous behaviors, EDA measurements and pain scales scores) and –5 h (EDA measurements and pain scales scores). Then, the dogs were subjected to a standardized trochleoplasty and general anesthesia. The time of tracheal extubation was defined as the time “zero” (T0) hour post-surgery. Video recording of the spontaneous behaviors was also performed at T24 and T48 post-surgery. Measurements of EDA and pain scales scores were recorded at T0.5, 1, 2, 3, 6, 9, 12, 24, 36, and 48 post-surgery. One observer (SAU), blinded to dog group attribution, performed live assessments in the following sequential order: VAS, 4A-VET, and EDA. Another observer (DVL) performed the video analysis of the spontaneous behaviors.

The dogs were randomized into three groups. Group 1 dogs (*n* = 10), received an oral placebo (Dextrose, Sigma-Aldrich Canada Ltd., Oakville, ON, Canada) between 3 to 2.5 h before T0 (*i.e.*, approximately 1.5 h before starting surgery), and the administration was repeated every 6 h until study completion. Group 2 dogs (*n* = 5) received a multimodal pre-emptive analgesia consisting of the following: 1) a transdermal fentanyl patch (2–3 µg/kg, Duragesic^TM^ 50, Janssen-Ortho Inc., Toronto, ON, Canada) applied to the skin 24 h prior to the surgery and maintained in place until study completion; 2) an epidural mixture injection of morphine sulfate (0.1 mg/kg, Morphine HP®25, Sandoz, QC, Canada) and ropivacaine (1 mg/kg Naropin^TM^ 0.2%, AstraZeneca Canada Inc., Mississauga, ON, Canada), administered 20 min prior to the surgery, followed by an 0.1 mg/kg epidural morphine sulfate injection given at 12, 24, and 36 h after extubation; 3) a subcutaneous (SC) tolfenamic acid injection (4 mg/kg Tolfedine^TM^ 4%, Vetoquinol Inc., Lure, France) administered 1 h prior to the surgery and repeated after 24 and 48 h; and 4) an oral administration of tramadol (10 mg/kg, V1002, Vetoquinol Inc., Lure, France) started 3 to 2.5 h prior to T0 and repeated every 6 h until study completion. Group 3 dogs (*n* = 10) received 10 mg/kg of tramadol orally between 3 to 2.5 h prior to T0 and every 6 h until study completion. The dogs in Groups 1 and 3 also received a sham or placebo for the transdermal, epidural, and subcutaneous administrations. Rescue analgesia (0.1 mg/kg hydromorphone intravenously [IV], 25–50 µg/h fentanyl patch, and 4 mg/kg SC tolfenamic acid) was provided if the VAS score exceeded 6.5 (out of 10), and/or the 4A-VET score exceeded 11 (out of a total score of 18).

At the end of the experiment at T54, all dogs were euthanized using an IV overdose of sodium pentobarbital (Euthanyl^TM^, Bimeda-MTC Animal Health Inc., Cambridge, ON, Canada).

### Anesthesia and Surgery Procedures

Anesthesia was induced with IV propofol to effect (up to 8 mg/kg, Propoflo^TM^ 1%, Abbott Animal Health, North Chicago, IL, USA). Lidocaine spray (10% w/w, Lidodan^TM^, Odan Laboratories Ltd., Pointe-Claire, QC, Canada) was administered onto the glottis prior to tracheal intubation. Volatile anesthesia was initiated with isoflurane (AErrane^TM^, Baxter Corporation, Mississauga, ON, Canada) in oxygen (oxygen flow originally set at 200 ml/kg/min and isoflurane vaporizer set at 3%) using a Bain coaxial system. Then, volatile anesthesia was maintained using mechanical ventilation set at a respiratory rate between 8–12 breaths/min and using a peak inspiratory pressure of less than 20 cm H_2_0 to achieve a constant end-tidal carbon dioxide of approximately 40 mmHg. End-tidal isoflurane was maintained at 1.7%. Lactated Ringer’s solution (Baxter Corporation, Toronto, ON, Canada) was IV-administered at a rate of 10 ml/kg/h throughout the anesthesia procedure. Cefazolin (25 mg/kg, Novopharm^TM^ Toronto, ON, Canada) was IV-administered 1 hour prior to surgery and repeated 6 to 8 hours up to the end of the study.

The standardized trochleoplasty was performed in the right femorotibial joint. A skin incision of 8 cm was made at the anterolateral aspect of the femorotibial joint. After incision of the articular capsule and medial stabilization of the patella, a rectangular abrasion (2×1 cm dimensions) trochleoplasty was performed in the right femoral trochlea. Next, the arthrotomy was sutured using 3–0 polydioxane absorbable sutures for the articular capsule, 3–0 polyglecaprone 25 polydioxanone absorbable sutures for the subcutaneous tissue, and 3–0 nylon sutures for the skin.

### Video Analysis of the Spontaneous Behaviors

We constructed a useful ethogram (Appendix S1) based on previous observations from pain research in the canine population [32], [33], personal observations and selection by a veterinary behaviorist (DFR). Behaviors were categorized using operational definitions. Categories were mutually exclusive and consisted of “Location in the kennel”, “Body position”, “Facial expression”, “Motor activity”, “Tail position”, and “Self-care”. The dogs were video-recorded during the same one-hour daylight period per session using a camera placed in front of the kennel. An automated video behavioral analysis system (The Observer^®^ XT, Noldus Information Technology, Tracksys Ltd., Nottingham, United Kingdom) was used to collect expression of spontaneous dog behaviors. Ten percent of the videos were selected randomly and reviewed by two independent observers (DVL, DFR). The occurrence rate of spontaneous behaviors was quantified for each video-recording session.

### Electrodermal Activity

EDA measures sympathetic response and is associated with pain and stress behavior [51], [52]. The portable device (Pain Gauge^®^, PHIS Inc., Dublin, OH, USA) converts electrical signals measured on the dry principal pad of the right thoracic limb to numerical values ranging from 0.1 (lowest value of stress and pain) to 9.9. Measurements of EDA were recorded in triplicate and averaged.

### Pain Scales

A VAS was used as a linear intensity pain scale with words that convey “no pain” (0-value) up to “worst pain” (100-value). The observer placed a mark along the line indicating the dog’s estimated pain intensity.

The composite 4A-VET pain scale recently tested by our group [48] was used again. It is composed of two sections. The first focuses on behavioral expressions of pain (4A-VETbeh) consisting of the “Global subjective appreciation”, “General attitude” and “Interactive behavior” subscales (Appendix S2). The second (4A-VETleg) includes orthopedic components of pain with “Gait evaluation”, “Reaction to handling of the surgical wound” and “Intensity of this reaction” subscales (Appendix S2). Each subscale scored pain intensity from 0 (no pain) to 3 (worst pain) and therefore, the total 4A-VET pain scale intensity ranged from 0 (no pain) to 18 (worst pain). Pain evaluation using the 4A-VET pain scale was performed in three successive and standardized phases: an initial, undisturbed observation of the dog, an interactive period of handling and encouragement and finally, a phase of systematic palpation of the incision and surrounding area of the operated leg.

### Statistical Analyses

The numbers and times of required rescue analgesia were described for each group. The data are reported as the median plus the 75^th^ percentile, unless otherwise specified.

The intra-observer reliabilities of the pain scales and repeatability of EDA were calculated based on the –96 h and –5 h evaluations using a weighted Cohen's kappa coefficient and Spearman’s rank correlations [53]. The inter-observer reliabilities of the video-recording spontaneous behavior assessment were calculated based on the 10% random set of spontaneous behavior changes using an intraclass correlation coefficient tested on log-transformed (to fulfill homoscedasticity and Wilk-Shapiro test normality requirements) data [54]. The internal consistency of the 4A-VET pain scale was assessed using a Cronbach's alpha coefficient [55].

Pain assessment scores were modeled over time using GEE for repeated measures [56], [57]. Data distribution was assessed and followed a negative binomial distribution (video analysis of spontaneous behavior), a Poisson distribution (VAS and 4A-VET pain scores and EDA measures) and a multinomial distribution (4A-VETbeh and 4A-VETleg scores). Model adequacy was verified using a thorough residual analysis [58]. For the negative binomial model, pairwise differences of mean estimates were expressed using estimated risk ratios along with a 95% confidence interval. To adjust for the multiple comparison tests performed, an adjusted-alpha level was set using the Bonferroni correction (original alpha-value divided by the number of comparisons of interest): 0.0041 for the video analysis of spontaneous behaviors (0.05/12), and 0.0015 for the EDA measurements and the pain scores (0.05/33).

To test the concurrent validity, multivariate GEE logistic regression models were used to assess the ability of each filmed spontaneous behavior to predict the pain scales scores. In addition, the regression models tested the following covariates: time, age, body weight and the use of rescue analgesia. The statistical significance of the above predictor variables and all of their possible dual interactions was tested with a stepwise-forward algorithm, using a threshold of *p* = 0.15 for including these factors in the multivariate model and a threshold of *p* = 0.20 for their removal [59]. A thorough residual analysis was performed for each model. The predictor variables showing clear non-linear relationships with the response variables were mathematically transformed to improve regression fit. Each final model was selected based on the best scatter of residuals over the regression line, coefficient of determination (R^2^) and quasi-likelihood information criterion [60]. The robust standard errors were calculated for all GEE estimates [61]. All analyses were conducted using a statistical software program (SAS system, version 9.2, SAS Institute Inc.); all tests were two-sided with an α threshold of 0.05.

## Supporting Information

Appendix S1 Ethogram of dog behaviors used for pain assessment.
**Spontaneous behaviors** were defined collegially by Pascale Rialland, Daphnée Veilleux-Lemieux, Diane Frank, Dominique Gauvin, and Eric Troncy. Behaviors were categorized using operational definitions. Categories were mutually exclusive and consisted of “Location in the kennel”, “Body position”, “Facial expression”, “Motor activity”, “Tail position”, and “Self-care”. The corresponding definitions are presented in the Appendix, as well as the Modifiers applicable to the different categories.
(DOCX)

Appendix S2 The 4A-VET pain scale.
The VETerinary Association for Animal Anesthesia and Analgesia (4A-VET) launched a composite multifactorial post-operative pain scale for dogs the 01/01/01. Originally created by Drs Patrick Verwaerde, Eric Troncy, Marc Gogny and Christophe Desbois, the 4A-VET pain scale had content validation by a panel of experts (Moens, Y.; Deschamps, J.-Y.; Cuvelliez, S.G., and Coppens, P.).
The canine 4A-VET post-operative pain scale is composed of two sections. The first focuses on behavioral expressions of pain (4A-VETbeh) consisting of the “Global subjective appreciation”, “General attitude” and “Interactive behavior” subscales. The second (4A-VETleg) includes orthopedic components of pain with “Gait evaluation”, “Reaction to handling of the surgical wound” and “Intensity of this reaction” subscales. Each subscale scores pain intensity from 0 (no pain) to 3 (worst pain) and therefore, the total 4A-VET pain scale intensity ranged from 0 (no pain) to 18 (worst pain).
(DOCX)
