# Supplementary material for: Validation of Orthopedic Postoperative Pain Assessment Methods for Dogs: A Prospective, Blinded, Randomized, Placebo-Controlled Study
Source: PLoS One. 2012 Nov 16;7(11):e49480. doi: 10.1371/journal.pone.0049480 (PMC3500314; doi:10.1371/journal.pone.0049480)
Supplement: Appendix S1 — Ethogram of dog behaviors used for pain assessment. Spontaneous behaviors were defined collegially by Pascale Rialland, Daphnée Veilleux-Lemieux, Diane Frank, Dominique Gauvin, and Eric Troncy. Behaviors were categorized using operational definitions. Categories were mutually exclusive and consisted of “Location in the kennel”, “Body position”, “Facial expression”, “Motor activity”, “Tail position”, and “Self-care”. The corresponding definitions are presented in the Appendix, as well as the Modifiers applicable to the different categories. (DOCX) [file pone.0049480.s001.docx]

**Appendix 1 –** Ethogram of dog behaviors used for pain assessment

**Behavioral Class 1: Location in the kennel**

Front cage The dog is physically in the front part of the cage (first half of the cage)

Back cage The dog is physically in the back of the cage (rear half of the cage)

**Behavioral Class 2: Body position**

Stand

Sit

Laying down

Other None of the above

Stand 2 pelvic Standing on pelvic limbs only

Body not visible Unable to see

**(*Weight bearing*, *Laying side* and *Pelvic limb* were described in modifiers)**

**Behavioral Class 3: Facial expression**

Not panting

Panting

Silent Mouth is closed

Bark Upward thrust of muzzle during apparent vocalization

Record once for each movement

Howling Muzzle directed dorsally, long-duration vocalization combining howl, yip, and bark in a continuous pattern

Yawn Mouth is fully opened with no vocalization

If done >1x in a row, record as separate events.

Yipping

Whine Audible whining accompanied by long expiratory efforts

Swallowing

Sniffing

Licking lips

Ears pulled back

Ears normal

Ears twitch Rapid movement of ears

Ears non-visible

Face non-visible

**Behavioral Class 4: Motor activity**

Immobilized

Jumps 2 paw or more not touching the ground

Walking

Running

Lame Animal is lame but has normal weight bearing

Shaking whole b Animal shaking whole body like wet dogs

Other None of the above

**(*Weight bearing* and head position were described in modifiers)**

**Behavioral Class 5: Tail position**

Tail up Tail is up in the air, above the continuation of the spine

Tail down Tail is down, below the continuation of the spine

Down tucked Tail down, tucked between the legs

**(*Tail* positions were described in modifiers)**

**Behavioral Class 6: Self-Care**

Scratch Repetitive pawing at body part with rear paw

Urinate

Defecate

Strain Time spent squatting unproductively

Vomit Physical expulsion of food or bile or unproductive attempts to do so

Careful position Dog carefully positioning itself in order to protect its wound

**Modifiers**

***Modifier Class 1: Weight bearing***

Full Full weight bearing

Partial Partial weight bearing

Not at all No weight bearing at all

Trampling, thoracic Switch from one side to the other on thoracic limbs

Trampling, pelvic Switch from one side to the other on pelvic limbs

***Modifier Class 2: Pelvic limb***

Missing Pelvic limb

Normal Equal repartition of weight on rear limbs

Unequal on Right Unequal repartition of weight on rear limbs (more weight on the right limb)

Unequal on Left Unequal repartition of weight on rear limbs (more weight on the left limb)

Alternate Switching from right to left

***Modifier Class 3: Laying side***

Missing Laying side

Right Right lateral

Left Left lateral

Dorsal

***Modifier Class 4: Head position***

Missing Head position

Head up

Head down

Head tilting

Head vertical

Looking around

Head shaking

Not visible

***Modifier Class 5: Tail movement***

Missing Tail movement

Tail moving

Tail immobile
