# Supplementary material for: Validation of Orthopedic Postoperative Pain Assessment Methods for Dogs: A Prospective, Blinded, Randomized, Placebo-Controlled Study
Source: PLoS One. 2012 Nov 16;7(11):e49480. doi: 10.1371/journal.pone.0049480 (PMC3500314; doi:10.1371/journal.pone.0049480)
Supplement: Appendix S2 — The 4A-VET pain scale. The VETerinary Association for Animal Anesthesia and Analgesia (4A-VET) launched a composite multifactorial post-operative pain scale for dogs the 01/01/01. Originally created by Drs Patrick Verwaerde, Eric Troncy, Marc Gogny and Christophe Desbois, the 4A-VET pain scale had content validation by a panel of experts (Moens, Y.; Deschamps, J.-Y.; Cuvelliez, S.G., and Coppens, P.). The canine 4A-VET post-operative pain scale is composed of two sections. The first focuses on behavioral expressions of pain (4A-VETbeh) consisting of the “Global subjective appreciation”, “General attitude” and “Interactive behavior” subscales. The second (4A-VETleg) includes orthopedic components of pain with “Gait evaluation”, “Reaction to handling of the surgical wound” and “Intensity of this reaction” subscales. Each subscale scores pain intensity from 0 (no pain) to 3 (worst pain) and therefore, the total 4A-VET pain scale intensity ranged from 0 (no pain) to 18 (worst pain). (DOCX) [file pone.0049480.s002.docx]

**Appendix 2 - The 4A-VET pain scale**

| Parameter | Definition | Score |
| --- | --- | --- |
| Global subjective appreciation | No pain  Light to moderate pain  Moderate to severe pain  Intolerable pain | 0  1  2  3 |
| General attitude | Among the following clinical signs, how many are present?  Respiratory modification, moaning, hunched back, stays immobile in antalgic posture, animal agitated or depressed, appetite decreased, and looks at, gnaws or licks wound |  |
|  | No sign present  1 sign only  2 to 4 signs present  5 and more are present | 0  1  2  3 |
| Interactive behavior | Animal is alert and responds to voice and touch  Responds timidly  Does not respond immediately  Does not respond or responds with aggressiveness | 0  1  2  3 |
| Gait evaluation | Normal use of limb  Limping but limb is used when walking  Limb used only at rest  No use of limb | 0  1  2  3 |
| Reaction to the surgical wound palpation | No visible or audible reaction after 4 palpations  Visible or audible reaction at the 4^th^ palpation  Visible or audible reaction at the 2^nd^ and 3^rd^ palpation  Visible or audible reaction at the 1^st^ palpation | 0  1  2  3 |
| Intensity of the reaction | No response  Answers slightly, tries to withdraw itself  Turns the head or vocalizes  Animal tries to flee or attack or is not assessable | 0  1  2  3 |

**TOTAL: _______ / 18**
